# Clear Aligner Extraction Treatment with Caterpillar Motion Staging: Biomechanical Rationale, Clinical Protocol, and Report of Two Cases

**DOI:** 10.3390/dj14040197

**Published:** 2026-03-31

**Authors:** David Martinez-Lozano, Carlos Rivero-Mourelle, Alberto-José López-Jiménez

**Affiliations:** 1 Department of Orthodontics and Dentofacial Orthopedics, Institución Mississippi University Center, 28010 Madrid, Spain; info@davidmlorthodonticeducation.com; 2 Department of Orthodontics and Dentofacial Orthopedics, La Salle University Center, 28023 Madrid, Spain; 3 Department of Dental Clincal Specilities , Complutense University of Madrid, 28040 Madrid, Spain; 4 School of Dentistry, Francisco de Vitoria University, 28880 Madrid, Spain

**Keywords:** invisible orthodontics, clear aligners, caterpillar motion, staging, orthodontics tooth movement, premolar extraction, case reports

## Abstract

**Background**: Closing extraction spaces with clear aligners remains a significant biomechanical challenge, frequently involving difficulties in sagittal control, torque expression, and intra-arch anchorage. Although various sequential or phased retraction strategies exist, the Caterpillar Motion protocol has not yet been formally defined. This clinical report describes the Caterpillar Motion staging protocol and illustrates its application through representative extraction cases, rather than providing a systematic review or experimental comparison. **Case Presentation**: Two adult patients with extraction-based malocclusions were treated using the Caterpillar Motion staging protocol. Case 1 involved bimaxillary first-premolar extractions with maximum anchorage requirements and periodontal limitations in the mandibular incisors. Case 2 presented as a full Class II malocclusion requiring maxillary first-premolar extractions with moderate anchorage for sagittal camouflage. In both cases, tooth movement was organized into alternating functional groups, with waves limited to 2 mm of sagittal closure. **Discussion**: The Caterpillar Motion protocol reduces the risk of aligner bowing effect, increases effective crown engagement, and redistributes anchorage demands by preventing simultaneous shortening of both arch extremities. Both cases demonstrated controlled anterior retraction, stable posterior anchorage, and favorable root parallelism. **Conclusions**: Caterpillar Motion offers a biomechanically coherent and clinically reproducible staging strategy for clear aligner extraction therapy. Further controlled studies are needed to validate its advantages over traditional linear and en-masse protocols.

## 1. Introduction

Clear aligner therapy has expanded rapidly over the last decade, enabling clinicians to manage increasingly complex malocclusions with removable appliances [1,2]. Among these challenges, first-premolar extraction cases remain one of the most demanding scenarios, primarily due to the difficulty of achieving controlled three-dimensional movement during sagittal space closure [3]. Multiple clinical studies have shown significant discrepancies between predicted and achieved movements in extraction cases—specifically affecting crown angulation, torque, and root parallelism—which highlights the limitations of current staging approaches [4,5,6]. Within clear aligner planning, staging is one of the most critical determinants of predictable tooth movement, as described in recent classifications of macro- and micro-staging principles [7]. Building on this, traditional linear staging approaches used in extraction cases—particularly en-masse anterior retraction—have been associated with unwanted effects such as uncontrolled tipping, anchorage loss, and bowing of the aligner, especially when large anterior segments are retracted simultaneously [8]. Recent finite element analyses reinforce these limitations by demonstrating how uniform loading across broad tooth groups generates unfavorable stress patterns, insufficient root control, and anchorage loss during space closure. These constraints have led to the exploration of progressive or staggered staging strategies, although such proposals often appear in isolation and without a cohesive biomechanical rationale [9,10]. Commercial extraction solutions—such as the Invisalign G6 protocol—represent attempts to standardize staging and torque control [11,12]. However, published evidence shows variable clinical predictability, persistent torque loss, and limited root uprighting despite the use of these systems [13,14,15]. Consequently, clinicians often resort to empirically developed modifications to improve staging efficiency. One of the clinical strategies that has gained broad adoption is the so-called Caterpillar Motion, a sequential and alternated approach to extraction-space closure. Despite its popularity, no formal definition, standardized methodology, or biomechanical justification has been published. Therefore, the aim of this clinical report is to formally define the Caterpillar Motion staging protocol, describe its conceptual and biomechanical foundations, and detail its clinical implementation in extraction-based clear aligner therapy. This protocol-oriented approach is illustrated through two representative adult cases with different anchorage demands, highlighting its applicability in complex clinical scenarios.

### 1.1. Background and Clinical Development of the Caterpillar Motion

The need to sequence extraction-space closure more intelligently in clear aligner therapy is not new; it has progressively developed over nearly two decades of clinical and biomechanical literature. Early reports of first-premolar extraction treatments with Invisalign, published in the mid-2000s, already highlighted persistent difficulties in achieving three-dimensional control during anterior retraction, with en-masse retraction showing a tendency to generate cumulative biomechanical side effects [16,17,18]. Although these initial clinical reports did not provide a systematic methodology, they clearly demonstrated that linear retraction of large anterior segments increased the likelihood of tipping, anchorage loss, and insufficient torque expression.

The first explicit attempt to introduce an alternated staging strategy was presented by Samoto and Vlaskalic in 2014, who proposed a system of “*waves*” in which different subgroups of teeth were retracted in an alternating motion. In their protocol, certain anterior teeth advanced while others remained temporarily stabilized, switching roles in successive phases. This staggered organization aimed to reduce the simultaneous load placed on the anterior segment and to improve root control during space closure [19].

Subsequently, Ojima et al. developed a clinical approach based on phased retraction, combining sequential planning, torque-control strategies, and auxiliary anchorage when necessary [20,21,22]. Their clinical pattern described a functional alternation between tooth groups during anterior retraction, which led many clinicians to refer to this movement pattern as “*Caterpillar Motion*”, due to its resemblance to the locomotion of a caterpillar—progressing forward by elevating certain “*legs*” while others provide support.

Chang et al. explicitly mentioned the term “*Caterpillar Motion*”, although without providing a formal definition or detailed protocol description. In their case reports, anterior retraction was divided into two consecutive phases as part of their clinical approach, aligning partially with the staggered logic proposed in earlier work [23]. Several clinical and finite element studies continued to explore various forms of staged retraction as an alternative to en-masse retraction. In most of these studies, staged retraction involved distalizing the canines first and only subsequently retracting the incisors as a unified anterior segment, without multiple alternations between groups [24,25,26,27]. Although this pattern does not fully correspond to the modern concept of Caterpillar Motion, it reinforces the principle that segmented retraction improves torque control, root angulation, and anchorage management.

Taken together, the historical evolution shows that Caterpillar Motion did not originate from a single author or publication but rather from the convergence of multiple clinical attempts to overcome the biomechanical limitations of linear staging. Despite partial references and conceptual approximations in the literature, a detailed description, clearly defined methodology, and complete systematization of the protocol remain absent.

### 1.2. Conceptual Definition of the Caterpillar Motion

The Caterpillar Motion is a protocol of sequential, alternated tooth movements designed to optimize three-dimensional control, improve the distribution of anchorage, and facilitate progressive root positioning during extraction-space closure with clear aligners. Its defining feature is that the subgroups of anterior teeth do not move simultaneously but instead alternate between phases of movement and phases of rest, creating a staggered, progressive pattern of advancement.

**Figure 1:**
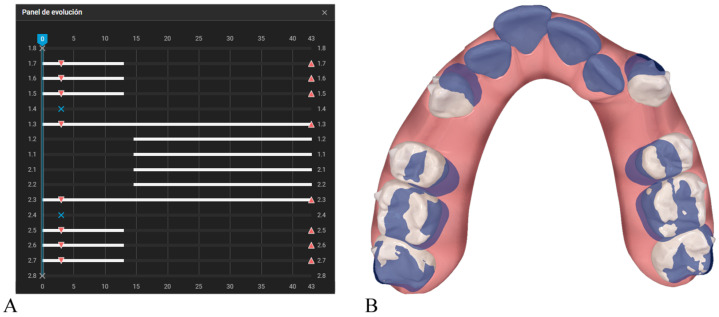
Invisalign^®^(Align Technology, San Jose, CA, USA) G6 protocol for maxillary first-premolar extractions with maximum anchorage. (**A**) Progress tracking tab. White bars indicate active tooth movement. Upward triangles represent attachment placement, downward triangles indicate attachment removal, and blue X symbols indicate tooth extraction. (**B**) Comparison of movements between aligner 1 (blue) and aligner 14 (white), corresponding to the completion of one third of the isolated canine retraction.

Among the sequencing protocols currently available in clinical practice, the most direct point of comparison is the Invisalign G6 protocol, which was developed specifically for maximum-anchorage first-premolar extraction cases. The G6 protocol allows for up to 5 mm of posterior anchorage loss when required by the case, and this mesial movement of the posterior teeth begins almost from the outset, occurring concurrently with the initial stages of canine retraction. The typical G6 sequence begins with the isolated retraction of the canines. Once approximately one-third of their movement has been achieved, the incisors are incorporated so that the remaining two-thirds of canine retraction are completed together with anterior retraction (Figure 1). Owing to its structure, the G6 is considered a segmented protocol, in which each tooth moves continuously during its designated phase; however, it is not an alternated protocol, as it does not involve repeated cycles in which one subgroup advances while another temporarily stabilizes.

In contrast, the Caterpillar Motion organizes space closure into alternated micro-sequences, distributing movement across phases that allow for a more controlled use of anchorage and a more balanced progression of the anterior segment. Its operational principles and clinical implementation are detailed in the following sections.

Although the Caterpillar Motion is primarily a sagittal sequencing protocol, a degree of conceptual confusion has persisted in the literature because alternation is also used in other staging strategies. Some authors have used the term “*frog pattern*” to refer to the Caterpillar Motion, yet this conflates two fundamentally different protocols [8]. Frog staging is an alternating sequence designed for vertical movements, particularly anterior intrusion [28,29], whereas the Caterpillar Motion employs alternation to control anteroposterior (sagittal) displacement, most notably in premolar extraction cases, although it can also be applied in the final phases of distalization. While both rely on alternation, they operate in distinct planes of movement and with different clinical objectives and therefore should not be considered interchangeable.

### 1.3. Clinical Staging Architecture and Programming

#### 1.3.1. Core Operational Pillars

The programming of the Caterpillar Motion is based on a carefully organized sequencing strategy that combines three operational pillars. These principles allow for precise control of sagittal tooth movement, prevent aligner deformation, and optimize anchorage distribution during extraction-space closure.

**Pillar 1—Simultaneous shortening of the arch’s terminal ends must be avoided.**

This principle establishes that the two terminal ends of the arch—the central incisors and the most distal molar—must not shorten at the same time.

If both ends shorten simultaneously: the aligner material loses structural integrity, the area corresponding to the extraction site becomes the region of lowest stiffness, and the aligner tends to bow, producing the characteristic bowing effect and deformation of the occlusal plane.

**Figure 2:**
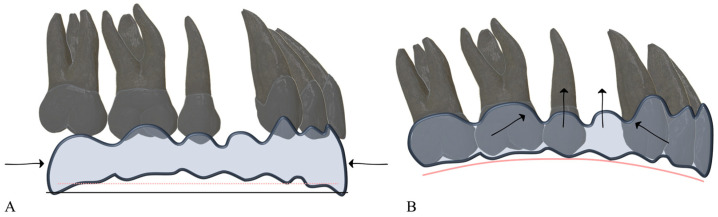
Bowing effect resulting from the simultaneous shortening of both ends of the arch. (**A**) Concurrent incisor retraction and mesialization of the second molars create an aligner length (dashed red line) shorter than the length of the dental arch (solid black line). Arrows indicate the simultaneous shortening at both ends of the arch. (**B**) When the aligner is seated on the arch, it “*bows*” due to insufficient rigidity, rendering it unable to maintain the integrity of the occlusal plane. Arrows illustrate the resulting displacement pattern, with anterior and posterior teeth moving toward the extraction space and vertical deformation occurring in the intermediate region.

The critical point is avoiding simultaneous retraction of the incisors and mesial movement of the most distal molar (commonly the second molar) (Figure 2). Maintaining the length of at least one terminal segment provides a stable resistance point that helps prevent aligner deformation.

**Pillar 2—Teeth with the highest individual anchorage value must not move simultaneously.**

In this protocol, “anchorage value” is operationally defined as the clinical resistance to displacement, a property directly correlated with the total root surface area and the specific distribution of the periodontal ligament available to dissipate orthodontic forces [30]. A tooth’s anchorage value results from a combination of factors such as root morphology, number of roots, and position within the arch. In the maxillary arch, for example, first molars (due to their multiple roots) and canines (due to their long roots) typically serve as the primary anchorage pillars. If teeth with high anchorage value move at the same time, arch stability decreases and unwanted displacements become more likely. Therefore, the clinician must identify which teeth provide the greatest anchorage and ensure that they do not move simultaneously within the sequencing.

**Pillar 3—Tooth movement is organized into functional groups.**

Since not all teeth can move simultaneously, they must be divided into functional groups whose movements will alternate throughout the protocol. The definition of these groups must follow the two previous pillars:

1. The teeth located at the two terminal ends of the arch cannot belong to the same group.
2. Teeth with the highest anchorage value must not be assigned to the same group.

Once the groups are defined, each will move or remain stable depending on the corresponding phase of the treatment.

#### 1.3.2. Structure of Waves (Sequential Phases)

A wave is a micro-sequence in which one tooth group moves while the other remains stable. In the Caterpillar Motion protocol, each active wave is typically planned to produce a limited amount of sagittal space closure. In the authors’ clinical experience, values around 2 mm per wave represent a practical and biomechanically manageable reference, allowing for adequate control of crown inclination and facilitating subsequent root repositioning during alternating phases. This value should not be interpreted as a strict cutoff but rather as a guideline that may be adjusted according to individual anatomical conditions, aligner material properties, and anchorage demands.

The duration of each wave depends directly on the activation per aligner, as determined by the planning software, which dictates how many aligners are required to complete those 2 mm. Once this distance is reached, the next wave begins by alternating the active tooth group.

#### 1.3.3. Representative Staging Configurations

The following examples illustrate how the three operational pillars of the Caterpillar Motion are expressed in the Progress-Evolution Panels commonly used in digital treatment-planning software. Each case demonstrates a different anchorage configuration and highlights the alternating sequencing that characterizes the protocol.

**Figure 3:**
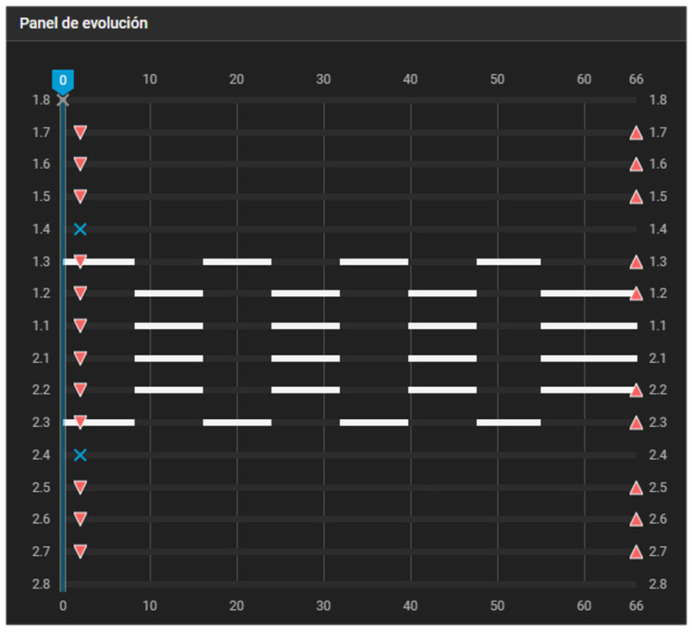
Caterpillar Motion configuration for first-premolar extraction with maximum anchorage. Alternating movements are programmed between the canines and incisors. White bars indicate active tooth movement. Upward triangles represent attachment placement, downward triangles indicate attachment removal, and blue X symbols indicate tooth extraction.

Figure 3 illustrates the classic case of first-premolar extraction managed with a maximum-anchorage configuration. If no movement of the posterior teeth is intended, the dentition is effectively divided into two independent single-tooth groups. Group 1 consists of the canines, whereas Group 2 comprises the incisors. In this scenario, adherence to the first pillar is not a concern, as the aligner will not be shortened at any distal endpoints at any stage of the process.

**Figure 4:**
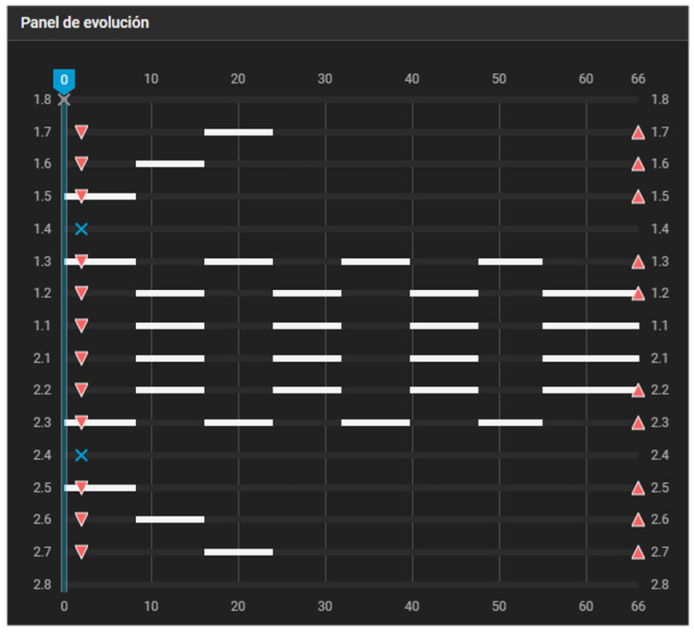
Caterpillar Motion configuration for first-premolar extraction with 2 mm of posterior anchorage loss. Group 1 consists of the canines, second premolars, and second molars, while Group 2 is composed of the incisors and first molars. Within Group 1, the second molars remain stationary until the second premolars have completed their programmed movement. White bars indicate active tooth movement. Upward triangles represent attachment placement, downward triangles indicate attachment removal, and blue X symbols indicate tooth extraction.

Figure 4 depicts a first-premolar extraction case with 2 mm of posterior anchorage loss. Because posterior tooth movement is required, the first two pillars become essential for defining the working groups. Group 1 consists of the canines, second premolars, and second molars, whereas Group 2 is composed of the incisors and first molars. Within Group 1, the second molars remain stationary until the second premolars have completed their mesial movement. By aligner #24, the mesial displacement of the posterior teeth had been completed, allowing for alternating movements of the incisors and canines to finish space closure.

**Figure 5:**
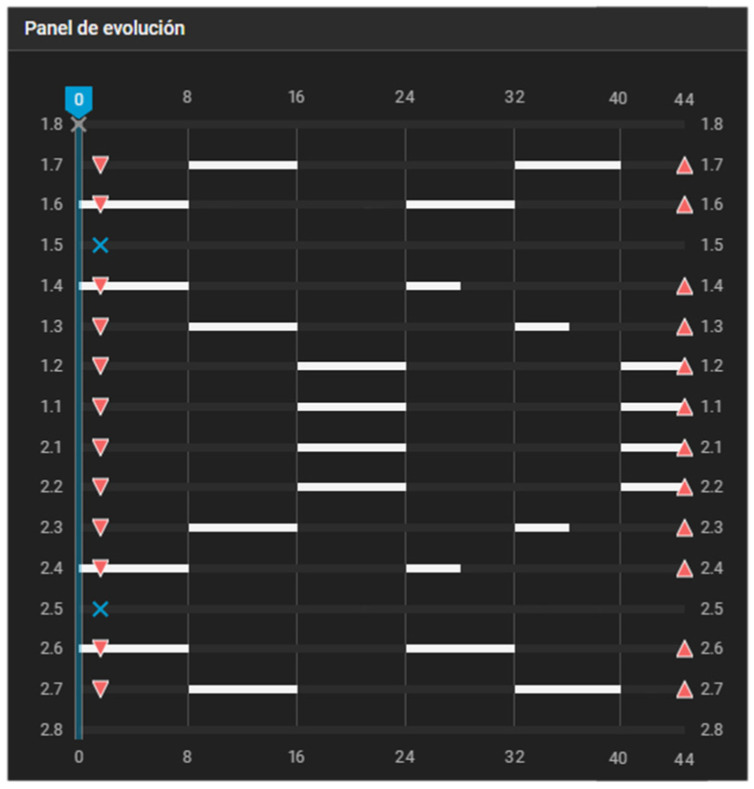
Caterpillar Motion configuration for second-premolar extraction, involving 4 mm of molar mesialization and 3 mm of anterior and first-premolar retraction. Group 1 consists of the first premolars and first molars, Group 2 comprises the canines and second molars, and Group 3 includes the incisors. During the first three waves, the teeth within each group will move 2 mm. By the fourth wave, the first molars will mesialize 2 mm, whereas the first premolars will advance only 1 mm until reaching their final position. In the fifth wave, the second molars will mesialize 2 mm, while the canines will retract only 1 mm. In the seventh wave, the incisors will retract 1 mm. White bars indicate active tooth movement. Upward triangles represent attachment placement, downward triangles indicate attachment removal, and blue X symbols indicate tooth extraction.

Figure 5 illustrates a second-premolar extraction case involving 4 mm of mesial molar movement and 3 mm of anterior and first-premolar retraction. Group 1 is composed of the first premolars and first molars, Group 2 comprises the canines and second molars, and Group 3 consists of the incisors.

## 2. Case Presentations

To illustrate the clinical implementation of Caterpillar Motion in extraction-based clear aligner therapy, we present two adult patients treated in a private orthodontic practice. Both cases involved premolar extraction space closure but differed in skeletal patterns and anchorage requirements: (1) a patient with severe mandibular crowding and bimaxillary first-premolar extractions managed with maximum anchorage, and (2) a patient with a full Class II malocclusion treated with maxillary first-premolar extractions and moderate anchorage for skeletal compensation.

In both cases, clear aligner therapy was planned according to the Caterpillar Motion principles described in the previous sections, with alternating staging of anterior and posterior segments and customized waves limited to 2 mm of sagittal space closure per active phase. All treatments were carried out by the same orthodontist using a standardized protocol.

### 2.1. Case 1—Severe Mandibular Crowding Treated with Four First-Premolar Extractions and Maximum Anchorage

#### 2.1.1. Diagnosis and Etiology

**Figure 6:**
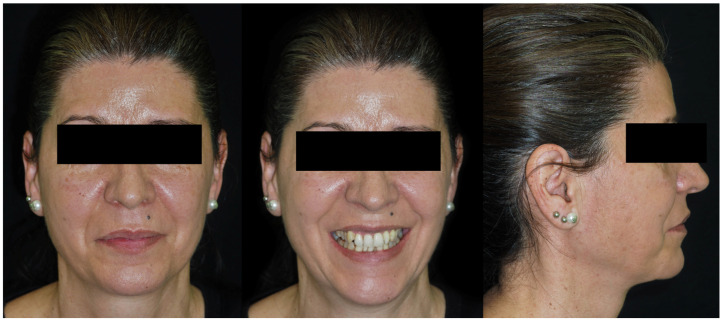
Initial facial photographic records of Clinical Case 1.

A 50-year-old female patient presented seeking improvement of her dento-facial aesthetics. Facial analysis showed balanced facial thirds, good symmetry, and a straight profile with a slight tendency toward bimaxillary protrusion (Figure 6). Mini-esthetic evaluation revealed a high smile line with gingival exposure of the right maxillary incisors, a convex incisal arc that did not contact the lower lip, and an upper dental midline centered on the facial midline but slightly tilted to the left. No parafunctional habits were reported.

**Figure 7:**
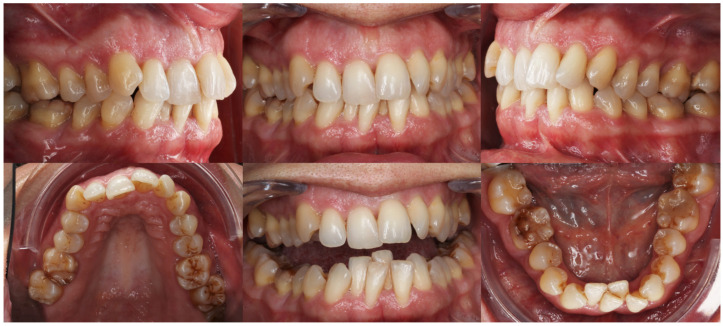
Initial intraoral records of Clinical Case 1.

Intraorally, the main finding was severe crowding in the mandibular anterior segment. Both arches exhibited dentoalveolar constriction, and the lower midline was deviated and inclined to the left relative to the upper midline. The overbite was increased with an accentuated mandibular curve of Spee, while the sagittal relationships were bilateral Class I (Figure 7).

**Figure 8:**
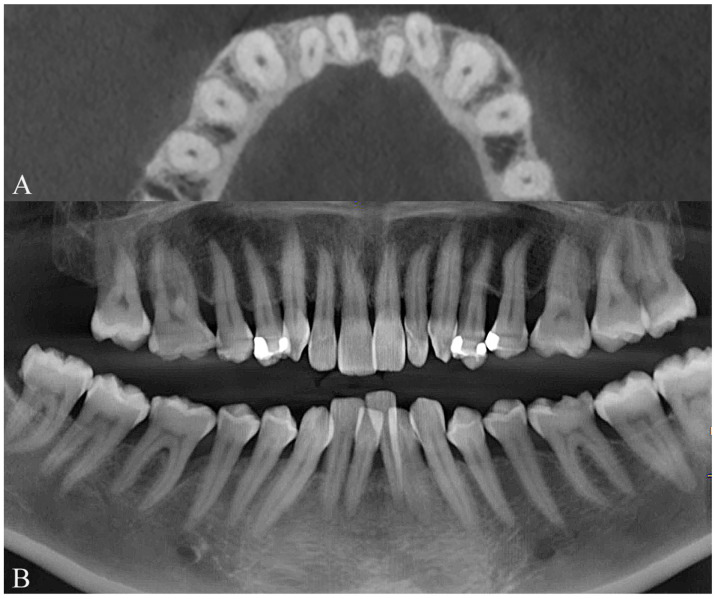
Initial radiographic records of Clinical Case 1. (**A**) CBCT sections of the mandibular anterior region. (**B**) Panoramic radiograph reconstructed from the CBCT.

**Figure 9:**
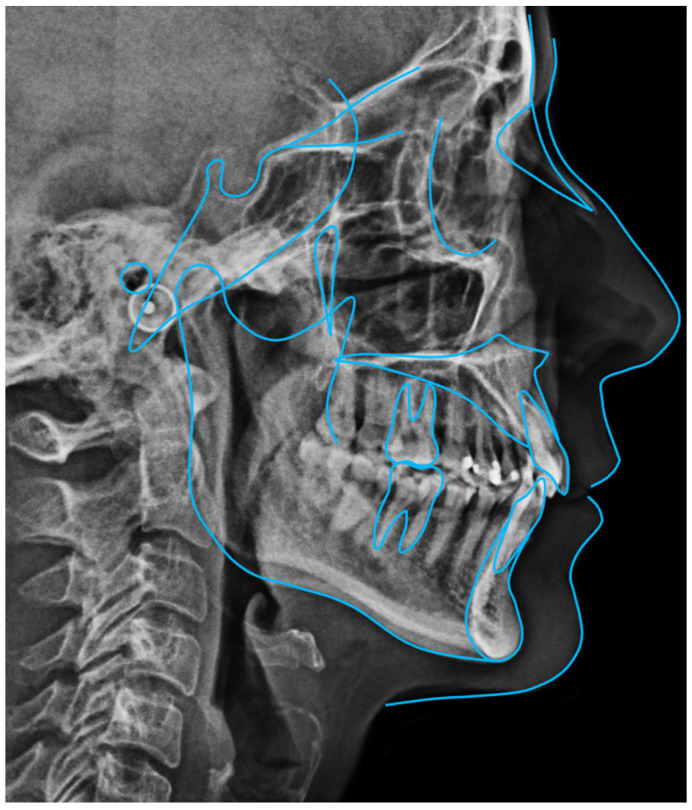
Initial cephalometric tracing of Clinical Case 1. The blue lines represent the cephalometric tracing overlaid on the radiographic image.

Because of the patient’s periodontal condition, a CBCT was obtained to evaluate the individual bony support. The axial slices showed a very thin mandibular symphysis and a bone defect between the lower central incisors (Figure 8A). The panoramic radiograph confirmed generalized bone loss and mesial tipping of the mandibular canines (Figure 8B). Cephalometric analysis revealed a skeletal Class I pattern with dolichofacial characteristics, retroclined maxillary incisors relative to the palatal plane, normally inclined mandibular incisors, and an increased interincisal angle (Figure 9).

#### 2.1.2. Treatment Objectives

The primary treatment objectives were to:

1. improve dental–facial aesthetics,
2. resolve the severe crowding by extracting all four first premolars,
3. center the dental midlines with the facial midline,
4. change both arches to a more parabolic form,
5. flatten the mandibular curve of Spee through lower anterior intrusion, and
6. avoid further deterioration of the periodontal condition.

Clear aligner therapy with the Invisalign^®^ system was selected after discussing the options with the patient.

#### 2.1.3. Digital Treatment Planning

Given the combination of severe lower crowding, reduced alveolar thickness, and the need for maximum anchorage, a Caterpillar Motion staging protocol was used.

Two functional groups were defined:

1. Group 1: canines and second premolars,
2. Group 2: incisors and first molars.

Second molars were programmed to move later in the staging together with the incisors but only with compressive movements that did not shorten the aligner.

**Figure 10:**
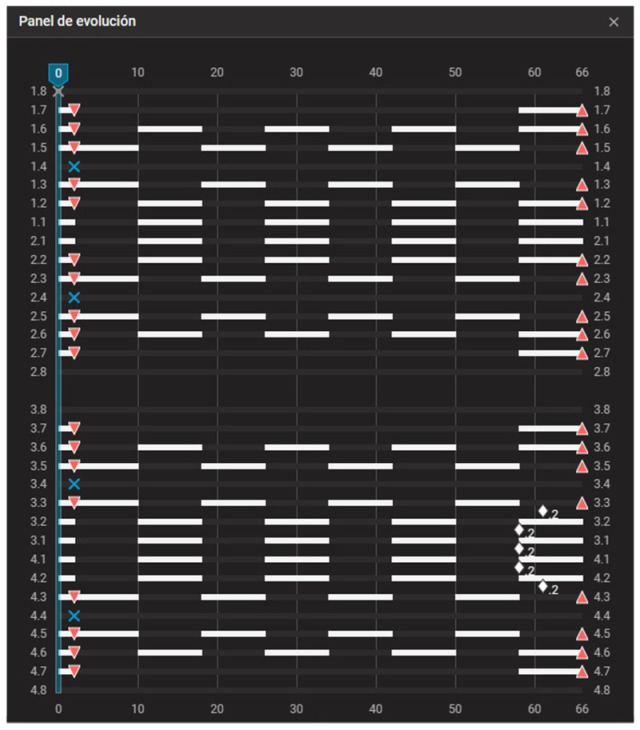
Staging panel of Clinical Case 1. A maximum-anchorage Caterpillar Motion protocol was used, alternating movements between the canines and incisors. White bars indicate active tooth movement. Upward triangles represent attachment placement, downward triangles indicate attachment removal, and blue X symbols indicate tooth extraction. White diamond symbols indicate interproximal enamel reduction (IPR), with the associated number representing the amount of reduction. Values displayed as “.2” correspond to 0.2 mm. The decimal format in the figure corresponds to the original display of the software and cannot be modified. To ensure clarity, an explanation has been added to the figure caption indicating that values such as “.2” represent 0.2 mm.

First-premolar extractions were planned at aligner 3. After extractions, Group 1 was moved into the extraction spaces over several aligners while Group 2 served as anchorage. In subsequent waves, Group 2 was retracted while Group 1 remained stable. This alternation continued until all extraction spaces were closed, following the Caterpillar Motion principles (Figure 10). Differential dental anchorage was used exclusively; no intermaxillary elastics or skeletal anchorage were employed.

**Figure 11:**
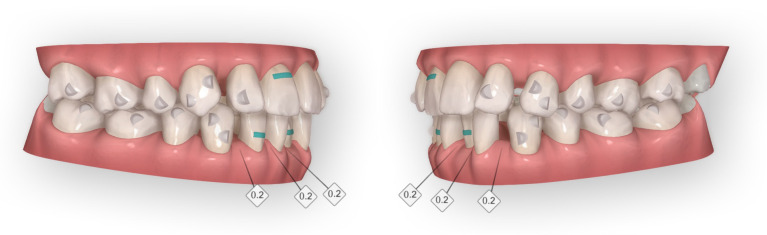
Smart Force features of Clinical Case 1. The optimized attachments of the Invisalign^®^ G6 protocol were used: optimized double retraction attachments on the canines and optimized anchorage-support attachments on the second premolars and molars. White diamond symbols indicate interproximal enamel reduction (IPR), with the associated numbers representing the amount of reduction (values correspond to 0.2 mm). The turquoise rectangular elements represent power ridges, corresponding to indentations in the aligner material designed to assist torque control.

Attachment selection followed the Invisalign G6 protocol. Optimized retraction attachments were placed on the canines to prevent uncontrolled distal tipping, and optimized maximum anchorage attachments were bonded to the second premolars and first and second molars to resist mesial drift. Maxillary lateral incisors received optimized root-control attachments, and power ridges were added to assist lingual root torque of the incisors (Figure 11). Optimized attachments were preferred over conventional vertical rectangular attachments because they provide sufficient control with less appliance retention—an advantage in a periodontally compromised patient—and adapt better at each aligner interface, reducing sharp edges and the need for additional elastics.

The first treatment phase consisted of 66 active aligners in each arch, with a 7-day wear protocol.

#### 2.1.4. Treatment Progress

**Figure 12:**
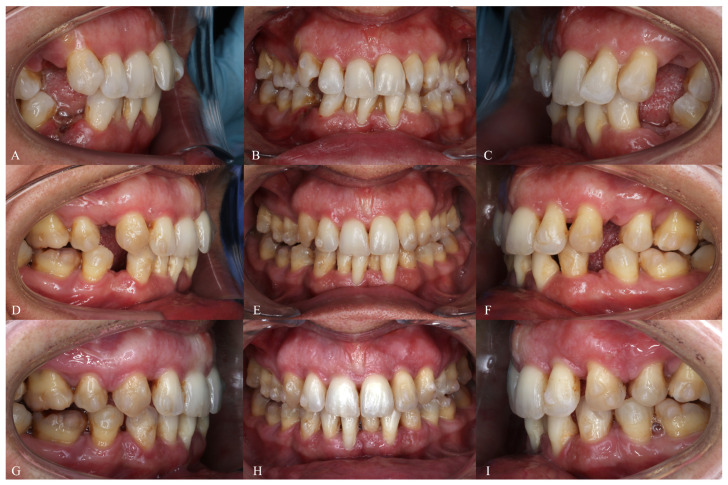
Treatment progress of Phase 1 in Clinical Case 1. (**A**–**C**) Aligner 3, showing attachment bonding and extraction of all four first premolars. (**D**–**F**) Aligner 23. (**G**–**I**) Aligner 47.

The patient was reviewed every six weeks to verify aligner fit, attachment integrity, oral hygiene, and compliance. The progressive closure of the extraction spaces and the alternation of the groups during the Caterpillar staging can be seen in the evolution of the aligners (Figure 12).

**Figure 13:**
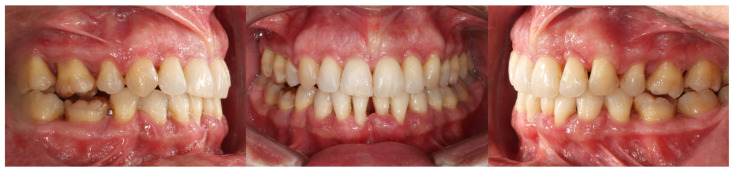
Photographs of the results of the first set of aligners in Clinical Case 1.

After completion of the initial 66 aligners, a new intraoral scan was obtained for refinement (Figure 13). The second phase consisted of 10 additional aligners, changed every 10 days, mainly to improve posterior contacts through controlled extrusion and to refine minor alignment details. At the end of active orthodontic treatment, the crowns of the mandibular first molars were restored and the black triangle between the lower central incisors was reduced with composite resin.

#### 2.1.5. Treatment Results

**Figure 14:**
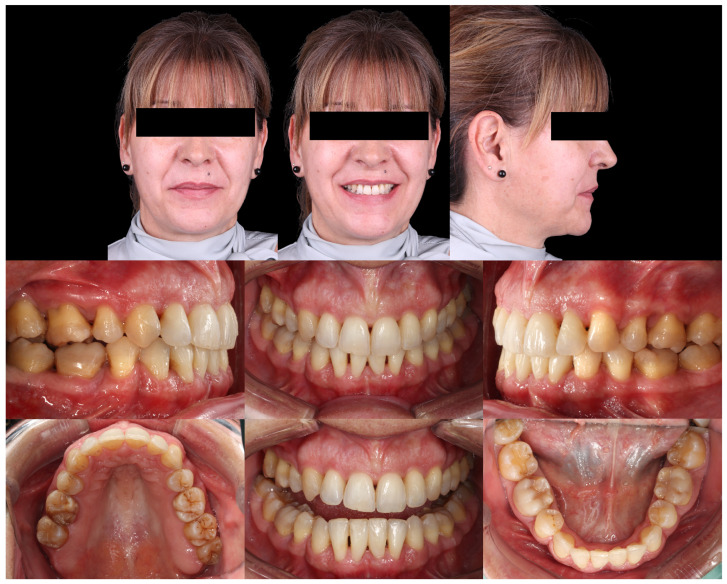
Final facial and intraoral photographic records of Clinical Case 1.

**Figure 15:**
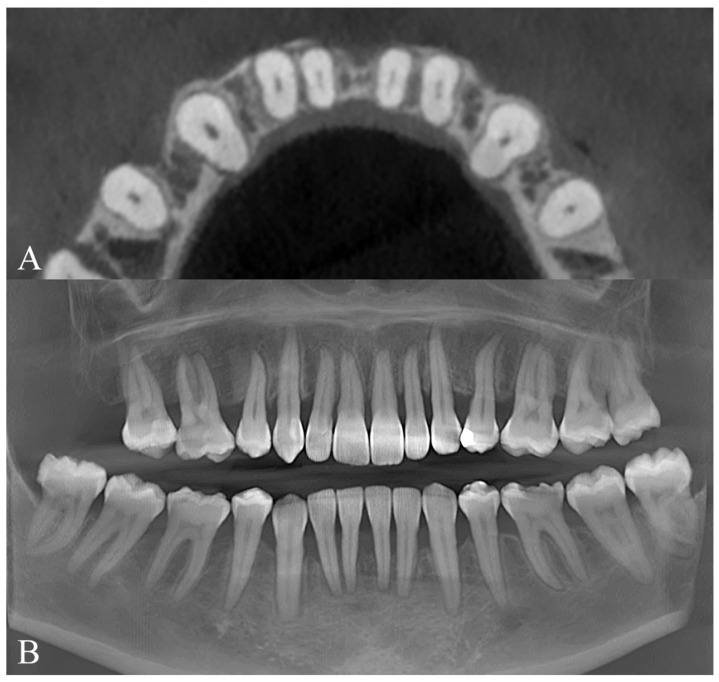
Final radiographic records of Clinical Case 1. (**A**) CBCT sections of the mandibular anterior region. (**B**) Panoramic radiograph reconstructed from the CBCT.

**Figure 16:**
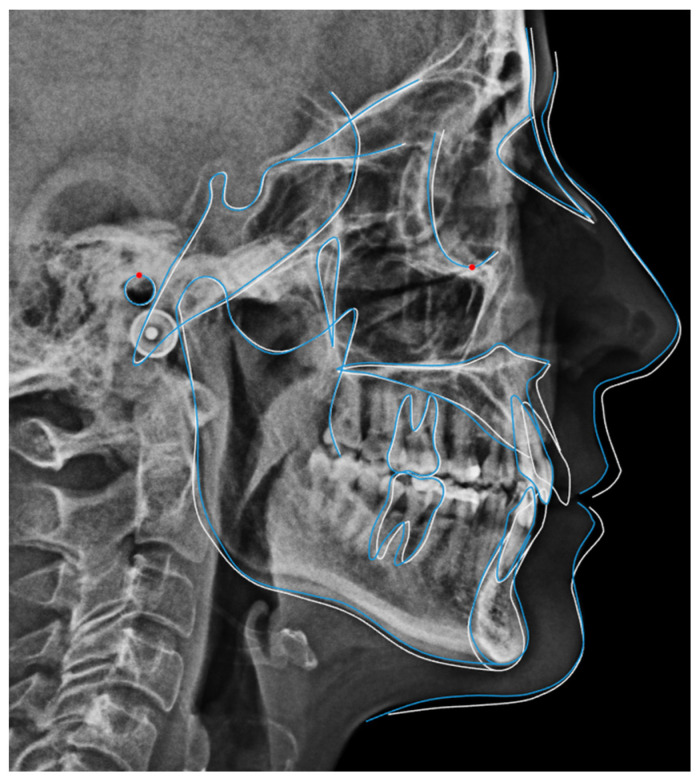
Cephalometric superimposition between the initial and final tracings of Clinical Case 1. The white lines represent the initial cephalometric tracing, while the blue lines correspond to the final tracing after treatment. Red dots indicate the reference landmarks used for superimposition, based on the Frankfurt horizontal plane.

The final extraoral and intraoral photographs demonstrate improvement of the smile aesthetics, resolution of the mandibular crowding, normalization of the curve of Spee, and well-coordinated arches with centered midlines (Figure 14). The final radiographs and cephalometric superimpositions (Figure 15 and Figure 16) show acceptable root parallelism, uprighting of the mandibular canines, and maintenance of the periodontal support in the mandibular incisors.

### 2.2. Case 2—Full Class II Malocclusion Treated with Upper First-Premolar Extractions and Moderate Anchorage for Skeletal Compensation

#### 2.2.1. Diagnosis and Etiology

**Figure 17:**
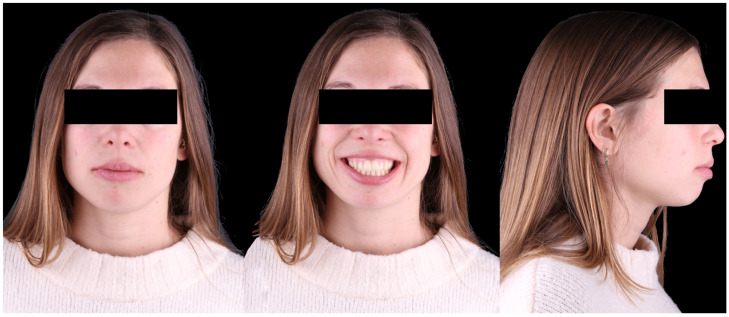
Initial facial photographic records of Clinical Case 2.

A 32-year-old female patient presented with the chief complaint of improving her lip seal and reducing ‘the distance between her anterior teeth.’ Facial analysis revealed balanced facial thirds, good symmetry, a mild occlusal cant with the right side slightly inferior, and a convex, protrusive profile (Figure 17). The mini-esthetic evaluation showed a high smile line with extensive gingival display, a convex smile arc parallel to the lower lip, and a deviation and inclination of the maxillary midline toward the left. No parafunctional habits were detected, except for lip incompetence at rest.

**Figure 18:**
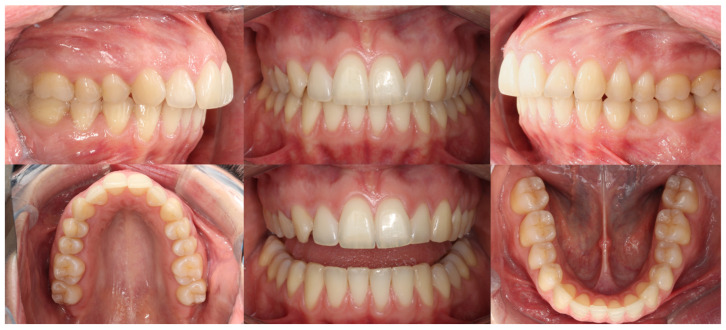
Initial intraoral records of Clinical Case 2.

Intraoral analysis revealed mild transverse dental constriction, a lack of coincidence between the maxillary and mandibular midlines, a bilateral full Class II relationship, increased overjet, gingival bulging associated with the mandibular incisors, and mild crowding in both arches (Figure 18).

**Figure 19:**
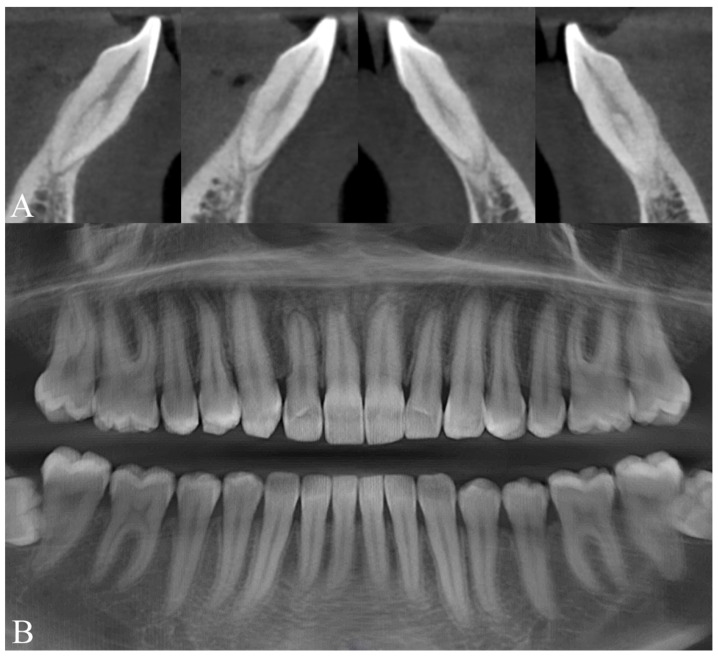
Initial radiographic records of Clinical Case 2. (**A**) CBCT sections of the mandibular anterior region. (**B**) Panoramic radiograph reconstructed from the CBCT.

**Figure 20:**
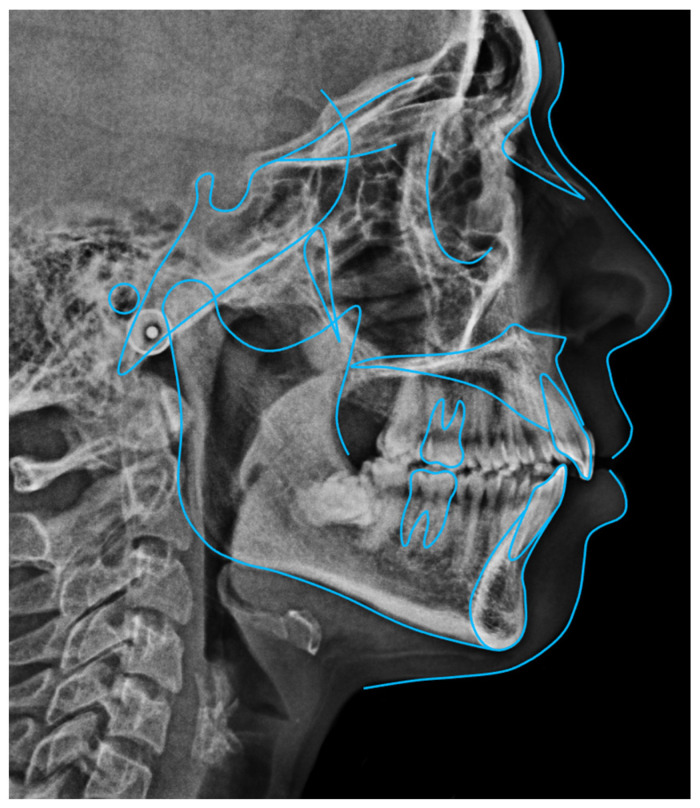
Initial cephalometric tracing of Clinical Case 2. The blue lines represent the cephalometric tracing overlaid on the radiographic image.

Radiographic examination showed maxillary teeth with minimal sagittal crown inclination and impacted mandibular third molars. CBCT sections of the incisors revealed thinning of the buccal cortical plate (Figure 19). Cephalometric analysis indicated a skeletal Class II pattern due to a short mandible, a mesofacial growth pattern, retroclined maxillary incisors, proclined mandibular incisors, and a normal interincisal angle (Figure 20).

#### 2.2.2. Treatment Objectives

The main treatment objectives were:

1. To compensate for the skeletal Class II discrepancy through extraction of the maxillary first premolars, establishing a Class I canine and premolar relationship and maintaining a full Class II molar relationship,
2. to reduce the overjet,
3. to improve lip seal, and
4. to reposition the mandibular incisors within the alveolar housing to minimize buccal periodontal risk.

Clear aligner therapy using the QuickSmile^®^ system was selected in agreement with the patient.

#### 2.2.3. Digital Treatment Planning

A medium-anchorage space-closure protocol was selected for the maxillary arch, consisting of 1 mm of posterior mesialization and 4 mm of anterior retraction. Because of inter-arch tooth-size discrepancies, the patient agreed to undergo restorative augmentation of the maxillary lateral incisors and canines; corresponding spaces were therefore planned in the final digital setup.

Staging followed a Caterpillar Motion protocol based on three functional groups:

1. Group 1: canines and second premolars.
2. Group 2: incisors and first molars.
3. Group 3: second molars.

**Figure 21:**
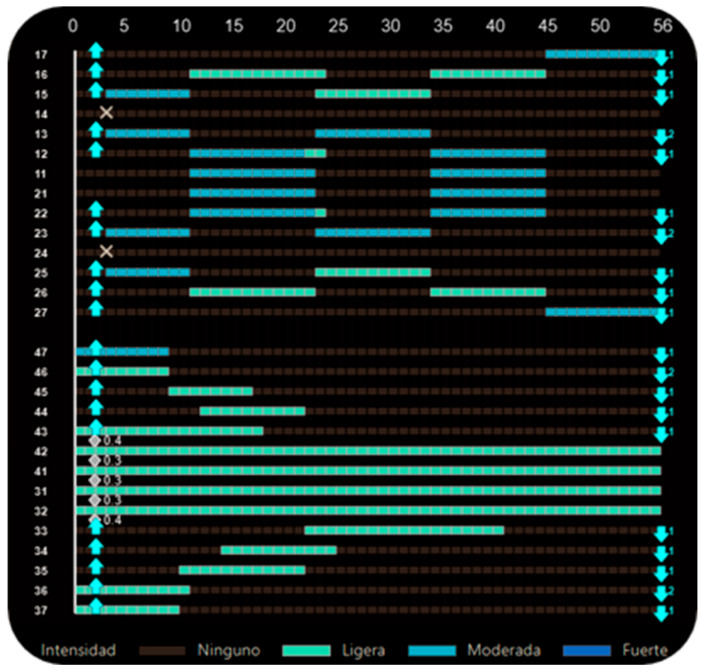
Staging panel of the first set of aligners in Clinical Case 2. A moderate-anchorage Caterpillar Motion protocol was used, alternating movements between the groups. Upward arrows indicate attachment placement, and downward arrows indicate attachment removal. Grey X symbols indicate extracted teeth. Grey diamond symbols with associated numbers represent interproximal enamel reduction (IPR). The colored bars indicate the intensity of tooth movement: light green represents mild movement, light blue represents moderate movement, dark blue represents strong movement, and grey indicates no movement.

Extraction of the first premolars was scheduled in aligner 1, and alternating movement patterns were implemented from the initial stage. Group 1 initiated space closure with a 2 mm reduction. Subsequently, movements alternated between Groups 1 and 2 until Group 2 reached its final position, after which Group 3 was activated to complete its staging (Figure 21).

**Figure 22:**
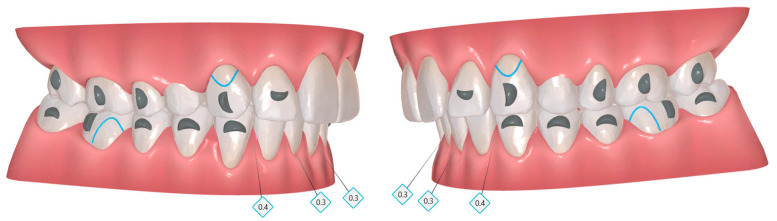
Attachments of Clinical Case 2. Vertical hemi-ellipsoidal attachments on maxillary canines, premolars, and molars, and horizontal hemi-ellipsoidal attachments on maxillary lateral incisors and on all mandibular teeth. The turquoise elements represent the attachment design. The blue line indicates an aligner button cutout.

Attachments features included vertical hemi-ellipsoidal attachments on maxillary canines, premolars, and molars to assist sagittal movements, and horizontal hemi-ellipsoidal attachments on maxillary lateral incisors and on all mandibular teeth to improve aligner fit (Figure 22). The patient wore Class II elastics (¼″, 4.5 oz) for 8–12 h per day as anchorage reinforcement, extending from the maxillary canines to the mandibular first molars.

The first treatment phase consisted of 56 active aligners per arch, with a 7-day wear protocol.

#### 2.2.4. Treatment Progress

**Figure 23:**
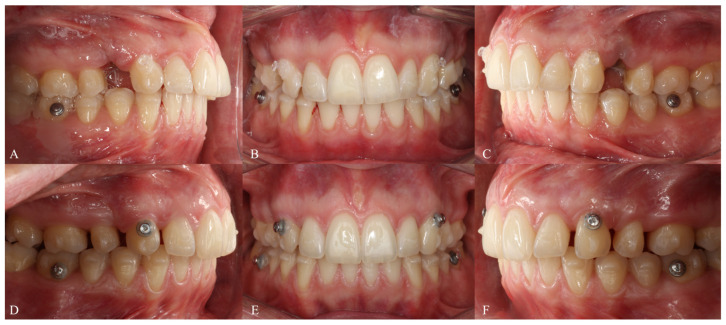
Treatment progress of Phase 1 in Clinical Case 2. (**A**–**C**) Aligner 3, showing attachment bonding and extraction of maxillary first premolars. (**D**–**F**) Aligner 30.

The patient was monitored every 6 weeks to assess aligner fit, compliance, and oral health. Maxillary space closure using the Caterpillar Motion protocol is illustrated in Figure 23.

**Figure 24:**
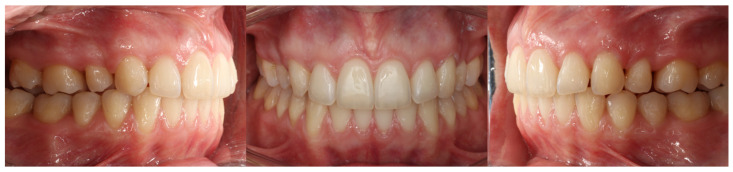
Photographs of the results of the first set of aligners in Clinical Case 2.

**Figure 25:**
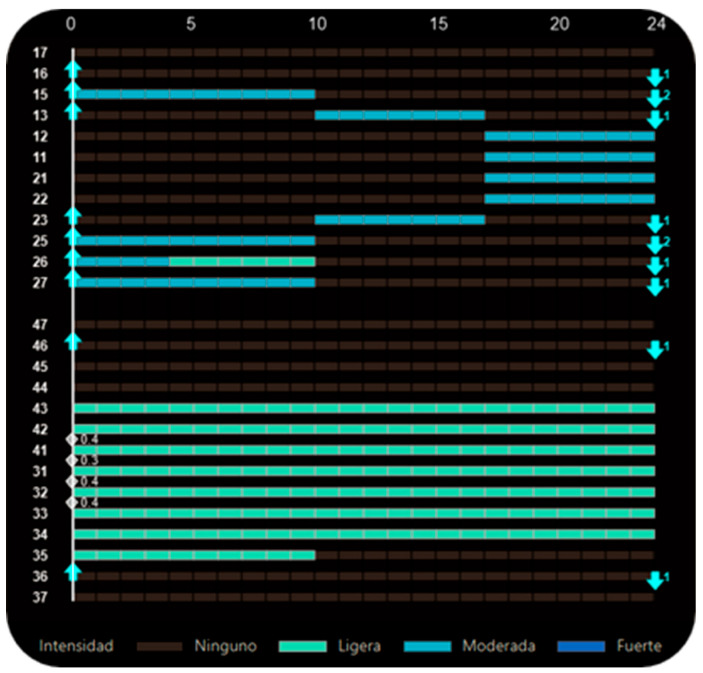
Staging panel of the second set of aligners in Clinical Case 2. A segmented staging approach was implemented, separating the movements of maxillary premolars, canines, and incisors. Upward arrows indicate attachment placement, and downward arrows indicate attachment removal. Grey diamond symbols with associated numbers represent interproximal enamel reduction (IPR). The colored bars indicate the intensity of tooth movement, ranging from no movement (grey) to light (green) and moderate (light blue) activation levels.

After completing the first set of aligners (Figure 24), the maxillary second premolars exhibited pronounced mesial crown tipping. Two refinement phases were therefore required, incorporating distal crown torque for these teeth and improving the posterior occlusal intercuspation to finalize the occlusion. A segmented staging approach was implemented, separating the movements of maxillary premolars, canines, and incisors (Figure 25). Class II elastics (¼″, 6 oz) were used for 12 h per day, extending from the maxillary second premolars to the mandibular first molars; during the second refinement, vertical posterior settling elastics were added.

Upon completion of the third aligner set, the clinical crowns of the maxillary lateral incisors and canines were restored.

#### 2.2.5. Treatment Results

**Figure 26:**
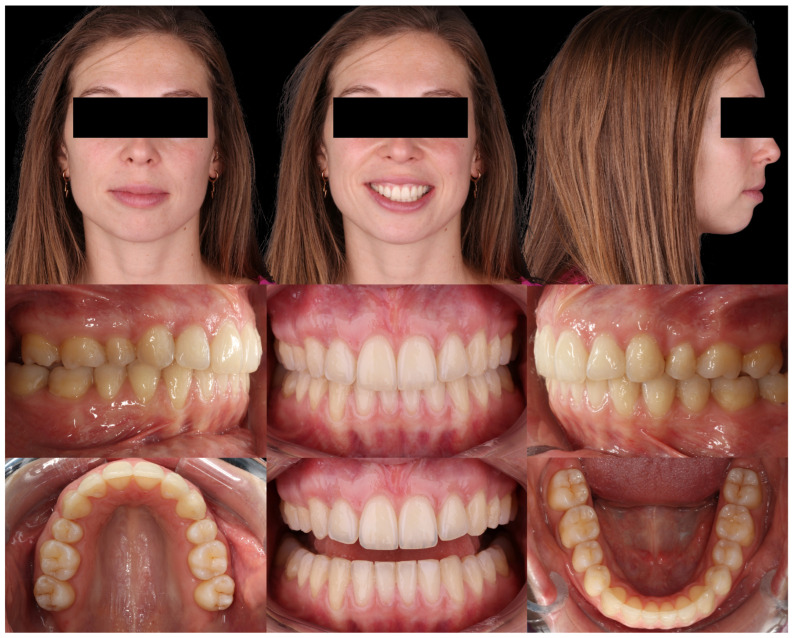
Final facial and intraoral photographic records of Clinical Case 2.

**Figure 27:**
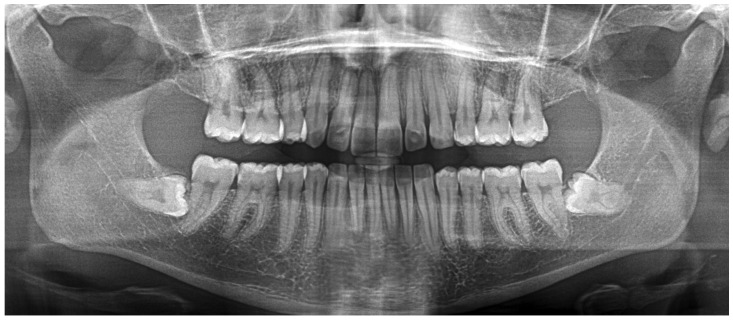
Final panoramic radiograph (prior to restorative procedures).

**Figure 28:**
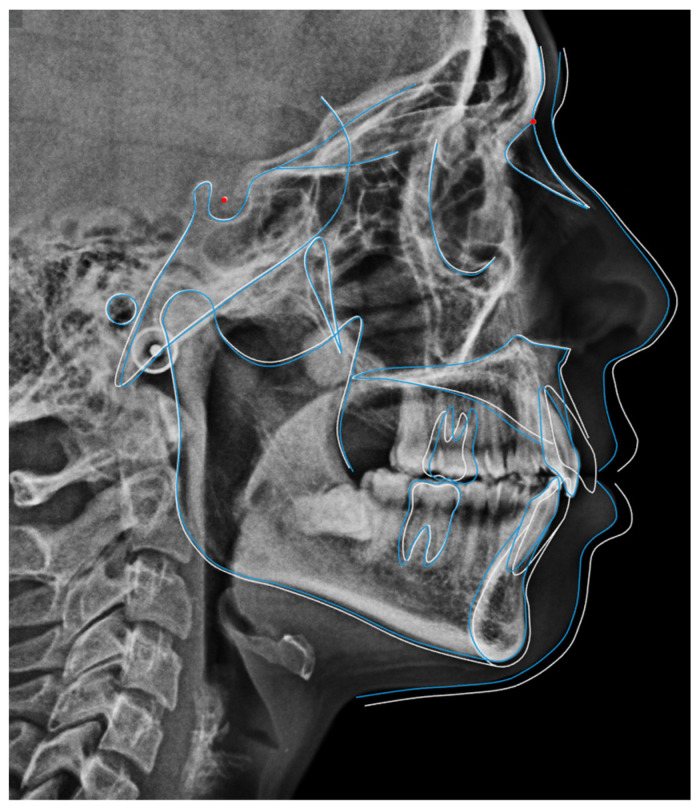
Cephalometric superimposition between the initial and final tracings of Clinical Case 2. The white lines represent the initial cephalometric tracing, while the blue lines correspond to the final tracing after treatment. Red dots indicate the reference landmarks used for superimposition, based on the Sella - Nasion plane.

Final extraoral and intraoral photographs show an improved facial profile, competent lip seal, and a stable, well-coordinated occlusion (Figure 26). Radiographs and cephalometric superimpositions obtained prior to restorative procedures demonstrate adequate root parallelism (except for the maxillary canines), reduced retroclination of the maxillary incisors, and favorable mandibular incisor inclination (Figure 27 and Figure 28).

## 3. Discussion

Clear aligner therapy has evolved substantially in managing extraction-based malocclusions; however, predictable sagittal control during space closure remains one of its most documented challenges. Traditional linear or en-masse retraction strategies frequently exhibit limitations regarding torque expression, anchorage management, and maintenance of the occlusal plane. The Caterpillar Motion protocol was developed as a clinically driven response to these constraints, introducing an alternating, wave-based staging architecture intended to improve biomechanical control during anterior and posterior movement.

The present article is the first to formally define this protocol, establish its conceptual pillars, describe its programming principles in detail, and illustrate its clinical execution through two representative extraction cases. The following discussion contextualizes these findings within current knowledge and highlights the biomechanical benefits, practical considerations, and remaining limitations associated with Caterpillar Motion staging.

### 3.1. Clinical and Biomechanical Implications

The Caterpillar Motion provides several biomechanical advantages that arise directly from its alternating staging.

#### 3.1.1. Reduction in the Bowing Effect and Enhanced Aligner Structural Stability

By preventing simultaneous shortening of the incisors and the most distal tooth in the arch, the Caterpillar Motion reduces the risk of aligner bowing, particularly in the extraction region, where the material exhibits its lowest stiffness. Maintaining the length of at least one arch extremity functions as a resistance point, stabilizing the aligner and reducing vertical deformation of the occlusal plane [31].

#### 3.1.2. Increased Effective Contact Area Between Aligner and Dental Crown

Organizing space closure into groups generates small interproximal gaps, allowing the aligner to fully engage the mesial, distal, buccal, palatal/lingual, and occlusal/incisal surfaces of the teeth involved in each active phase. This enhanced three-dimensional engagement improves the functional fit of the aligner and supports more precise control of tooth movement [19].

#### 3.1.3. Improved Intra-Arch Anchorage Distribution

By avoiding simultaneous activation of teeth with the highest individual anchorage value, the Caterpillar Motion promotes a more balanced distribution of anchorage. Teeth that remain passive during each wave act as functional anchorage, stabilizing the arch and reducing the risk of unwanted molar mesialization or sagittal loss of control during anterior retraction [32].

#### 3.1.4. Rest Intervals and Progressive Root Positioning

The alternation between active and passive phases introduces biomechanical rest intervals that allow for recovery of root parallelism, dissipation of accumulated stresses, and more favorable progressive repositioning of the roots within the alveolus. These pauses reduce the accumulation of uncontrolled tipping and promote more physiological loading patterns for the periodontal ligament [3].

### 3.2. Clinical Considerations and Pitfalls

#### 3.2.1. Confusing “Shortening Both Ends of the Arch” with “Moving Teeth at Both Ends of the Arch”

The pillar prohibiting simultaneous shortening refers specifically to retracting the incisors while mesializing the most distal molar, as both actions reduce the functional length of the aligner. Some clinicians mistakenly interpret this as forbidding any posterior movement. Molars can be expanded, rotated, compressed, or intruded without compromising biomechanics, as long as they do not shorten the arch during anterior retraction.

#### 3.2.2. Excessive Activations

Individual aligner activations greater than 0.25 mm increase the risk of uncontrolled tipping, hinder full expression of programmed movements, and reduce the capacity for root correction during passive phases. Activations near 0.20 mm tend to be more stable and physiologic.

#### 3.2.3. Accelerating Aligner Changes Without Biomechanical Justification

Although some clinicians propose shortened wear intervals (e.g., every 5 days), this reduces effective expression time, increases the likelihood of tracking issues, and limits root correction. In most cases, a 7-day change protocol provides a safer balance between efficiency and biomechanical stability.

#### 3.2.4. Monitoring Inter-Arch Coordination During Space Closure

In cases involving bimaxillary premolar extractions, the clinician must ensure that the rate of space closure in the maxillary arch does not exceed that of the mandibular arch. If overjet is consumed too rapidly, incisal interferences, premature anterior contact, or posterior open bite may occur. When this occurs, the maxillary space-closure rate must be slowed, or its sequencing further staggered, to maintain proper inter-arch coordination throughout retraction.

### 3.3. Limitations and Future Directions

Although the Caterpillar Motion offers conceptual and biomechanical advantages, several limitations must be acknowledged. First, this protocol relies heavily on precise digital staging and assumes that the programmed alternation is accurately transferred to the clinical environment. Any deviation in aligner fit, patient compliance, or attachment integrity may alter the intended sequence and reduce its effectiveness. Second, because movement is distributed across staggered micro-phases treatment time may increase in comparison with more linear staging approaches, particularly in complex extraction cases requiring multiple refinements. Third, the protocol’s success depends on the clinician’s ability to correctly define functional groups and estimate anchorage values, steps that remain partly subjective and operator-dependent. Moreover, high-quality scientific evidence on Caterpillar Motion remains scarce. Most data currently available derive from clinical experience, case reports, or extrapolated findings from finite element analyses of staggered or segmented retraction. While a direct comparative table with other established protocols (such as Invisalign G6) would provide additional clarity, we believe that a rigorous clinical comparison requires specific prospective data on tooth movement efficiency and roots parallelism that go beyond the descriptive scope of this article. Therefore, prospective controlled studies comparing Caterpillar Motion with G6 protocols, conventional staging, or alternative sequencing strategies are needed to determine its true clinical superiority. Additional research should also focus on understanding how aligner material behavior, attachment design, and differential root anatomy interact with alternating staging, as well as exploring artificial-intelligence-assisted staging tools that could automate or optimize wave design.

## 4. Conclusions

The Caterpillar Motion protocol provides a structured and biomechanically coherent approach to managing extraction-space closure with clear aligners. By organizing tooth movement into alternating functional groups and avoiding concurrent shortening of both arch extremities, the protocol enhances sagittal control, optimizes intra-arch anchorage distribution, and reduces the risk of aligner deformation. The two clinical cases presented demonstrate its applicability across varying anchorage requirements and skeletal patterns. Although further controlled studies are required to validate its superiority over traditional staging methods, the Caterpillar Motion represents a reproducible and practical framework for improving predictability in clear aligner extraction therapy.

## Data Availability

The original contributions presented in this study are included in the article. Further inquiries can be directed to the corresponding author.
